# On the robustness of detective quantum efficiency within the limits of IEC 61267 RQA standard radiation qualities

**DOI:** 10.1093/rpd/ncae029

**Published:** 2024-02-26

**Authors:** Stefan Pojtinger

**Affiliations:** Physikalisch-Technische Bundesanstalt (PTB), National Metrology Institute, Bundesallee 100, Braunschweig D-38116, Germany

## Abstract

IEC 61267 allows a certain leeway regarding the establishment of radiation qualities in order to enable the use of X-ray tubes having different anode angles and inherent filtrations. This allowance has a direct impact on the calculation of the detective quantum efficiency and may potentially complicate any comparison of different imaging detectors based on this quantity. This work investigates this effect by applying computational methods. To this end, an algorithm was implemented to calculate the variation of the squared signal-to-noise ratio per air kerma for RQA standard radiation qualities and to deduce corresponding uncertainties based on GUM Supplement 2. For RQA standard radiation qualities, the results show standard uncertainties for the squared signal-to-noise ratio per air kerma of between 0.05 and 2.1%. Comparing imaging detectors based on detective quantum efficiency is associated with substantial uncertainty for some radiation qualities. This is due to the different photon fluences with respect to energy that are allowed by IEC 61267 for identical standard radiation qualities.

## Introduction

The detective quantum efficiency $(DQE)$ characterises the intrinsic dose efficiency of an imaging detector under test conditions typically found in test laboratories or in manufacturing facilities. It is applied for imaging detectors used in radiographic imaging (IEC 62220-1-1), mammography (IEC 62220-1-2) and dynamic imaging (IEC 62220-1-3)^(^^1–3^^)^. Currently, $DQE$ is considered the most important figure of merit for characterising the physical imaging performance of X-ray detectors and it is widely used in scientific publications as well as in marketing materials and marketing datasheets. To calculate $DQE$, the squared signal-to-noise ratio per air kerma $\left({SNR}_{\mathrm{in}}^2\right)$ must be known. This can be calculated for any radiation quality based on the corresponding photon fluence with respect to energy $\left({\phi}_E\right)$.

IEC 62220-1^(^^1–3^^)^ gives the mandatory values of ${SNR}_{\mathrm{in}}^2$ for standard radiation qualities as described in IEC 61267^(^^4^^)^, these being defined by specific X-ray tube voltages (in terms of practical peak voltage, $PPV$), filtrations and a normative procedure for the generation of standard radiation qualities. To stay within the requirements of IEC 61267^(^^4^^)^, the achieved half-value layers ($HVL$s) must be measured and compared with those given in the standard for validation.

On the other hand, IEC 61267^(^^4^^)^ allows some leeway regarding the establishment of radiation qualities in order to enable the use of X-ray tubes with different anode angles and inherent filtrations, and this can lead to different values for ${\phi}_E$ for the same radiation quality. Also, ${\phi}_E$ can differ for the same standard radiation quality established within the requirements of IEC 61267^(^^4^^)^ because of the uncertainty associated with $PPV$ measurements. The hypothesis of this work is that this can lead to different values of ${SNR}_{\mathrm{in}}^2$ for the same standard radiation quality and thus complicate the comparison of different imaging detectors based on $DQE$.

Another motivation for the investigation conducted in this work is that the procedure as currently published in IEC 62220-1^(^^1–3^^)^ for calculating the ${SNR}_{\mathrm{in}}^2$ values was never made publicly available and is based on personal communication and unpublished algorithms.

To tackle both challenges, this work sought to systematically investigate the variation of ${SNR}_{\mathrm{in}}^2$ within a specific standard radiation quality and lay the foundation for the planned inclusion of updated ${SNR}_{\mathrm{in}}^2$ values within IEC 61267^(^^4^^)^ for RQA standard radiation qualities. Special emphasis was placed on providing a clear presentation of the methodology for calculating these values.

## Methods

The principal approach of this work was to computationally calculate ${\phi}_E$ for a range of different anode angles and $PPV$s. For each such computationally calculated ${\phi}_E$, the additional filtration for establishing the corresponding RQR radiation quality was determined by an algorithm and then used to calculate the corresponding photon fluence for the linked RQA radiation quality, which, in turn, was used to calculate ${SNR}_{\mathrm{in}}^2$.

### Detective quantum efficiency and signal-to-noise ratio

${SNR}_{\mathrm{in}}^2$ is the squared signal-to-noise ratio per air kerma in the units of ${\mathrm{mm}}^{-2}\ \mathrm{\mu} \mathrm{Gy}$. For an air kerma ${K}_{\mathrm{air}}$, an energy $E$ and ${\phi}_E$, ${SNR}_{\mathrm{in}}^2$ can be calculated as


(1) \begin{equation*} {SNR}_{\mathrm{in}}^2=\frac{\int{\phi}_E\ \mathrm{d}E\ }{K_{\mathrm{air}}} \end{equation*}

This quantity is defined in the international standards IEC 62220-1-1, IEC 62220-1-2 and IEC 62220-1-3 ^(^^1–3^^)^ to calculate $DQE$ as follows:


(2) \begin{equation*} DQE={MTF}^2\frac{K_{\mathrm{air},\kern0.1em\mathrm{test}}\ {SNR}_{\mathrm{in}}^2}{W_{\mathrm{out},\kern0.1em \mathrm{corrected}}} ,\end{equation*}

where $MTF$ is the pre-sampling modulation transfer function, ${K}_{\mathrm{air},\mathrm{test}}$ the air kerma and ${W}_{\mathrm{out},\kern0.75em \mathrm{corrected}}$ the noise power spectrum corrected for lag effects for an image acquisition under investigation.

### Calculation of ${\boldsymbol{SNR}}_{\mathbf{in}}^{\mathbf{2}}$

As seen in Equation 1, ${SNR}_{\mathrm{in}}^2$ can be calculated based on a photon fluence with respect to the energy ${\phi}_E$ and a corresponding air kerma ${K}_{\mathrm{air}}$. For a specific photon fluence ${\phi}_E$, ${K}_{\mathrm{air}}$ can also be derived from ${\phi}_E$ by calculating the expected value of $E{\left(\frac{\mu_{\mathrm{tr}}}{\rho }(E)\right)}_{\mathrm{air}}$, with the mass energy-transfer coefficients for air ${\left(\frac{\mu_{\mathrm{tr}}}{\rho }(E)\right)}_{\mathrm{air}}$:


(3) \begin{equation*} {K}_{\mathrm{air}}=\int{\phi}_EE\ {\left(\frac{\mu_{\mathrm{tr}}}{\rho }(E)\right)}_{\mathrm{air}}\mathrm{d}E \end{equation*}

Therefore, only ${\phi}_E$ and ${\left(\frac{\mu_{\mathrm{tr}}}{\rho }(E)\right)}_{\mathrm{air}}$ are needed for the calculation of ${SNR}_{\mathrm{in}}^2$. Values for $\left(\frac{\mu_{\mathrm{tr}}}{\rho }(E)\right)$ were derived from Monte Carlo simulations using the Monte Carlo Framework EGSnrc^(^^5^^)^. The usercode g was used for a direct calculation of ${\left(\frac{\mu_{\mathrm{tr}}}{\rho }(E)\right)}_{\mathrm{air}}$ for a range of energies. The settings for the Monte Carlo simulations are shown in Table 1. Calculations were performed for 2000 energies in the range of $0.001$ to $1\ \mathrm{MeV}$ on a logarithmic scale for a Monte Carlo variance of <$0.002\%$.

**Table 1:** EGSnrc simulation parameters for the calculation of $\left(\frac{\mu_{tr}}{\rho }(E)\right)$.

EGSnrc simulation parameters	Settings for the calculation of $\left(\frac{\mu_{\mathrm{tr}}}{\rho }(E)\right)$
ECUT	0.512 MeV
Global PCUT	0.001 MeV
Atomic relaxations	On
Photon cross sections	Mcdf-xcom [6]
Density correction file	Air_dry_nearsealevel
Bremsstrahlung correction	NRC

For the calculations of ${SNR}_{\mathrm{in}}^2$, the PTB inhouse Python module bueSpec was used, which performs the calculations as described by Equations 1 and 3. Therefore, ${SNR}_{\mathrm{in}}^2$ values were interpolated quadratically on a log–log scale and the integration was performed by Simpson’s rule. The lower energy cutoff for these calculations was set to $0.001\ \mathrm{MeV}$.

### Computationally calculated RQR standard radiation qualities

The software toolkit SpekPy^(^^7^^)^ was used to calculate ${\phi}_E$ as a function of the anode angle and $PPV$ at 1 m distance (air). For all calculations, SpekPy was used with the default configuration applied.

With ${\phi}_E$ as an input, bueSpec was used to determine the additional aluminum filtration needed for the establishment of computational RQR qualities. Based on the Beer–Lambert law, an attenuated value for the air kerma ${K}_{\mathrm{air},\mathrm{att}}$ was calculated in dependence of an aluminum thickness ${t}_{\mathrm{al}}$:


(4) \begin{equation*} {K}_{\mathrm{air},\kern0.1em\mathrm{att}}=\int{\phi}_E\ {e}^{-{\mu}_{\mathrm{al}}\ {t}_{\mathrm{al}}}\ E\ {\left(\frac{\mu_{\mathrm{tr}}}{\rho }(E)\right)}_{\mathrm{air}}\mathrm{d}E ,\end{equation*}

where ${\mu}_{\mathrm{al}}$ is the total attenuation coefficient including coherent scattering, as published in the XCOM database^(^^8^^)^. In a first step, Equation 6 was used to implement a numerical procedure capable of calculating $HVL\left(\ {\phi}_E\right)$ for aluminum by solving the following equation:


(5) \begin{equation*} {K}_{\mathrm{air}}-2\ {K}_{\mathrm{air},\kern0.1em\mathrm{att}}\left({t}_{\mathrm{al}}\right)=0, \end{equation*}

in which case ${t}_{\mathrm{al}}$ becomes the $HVL$. For this, the Python optimizer scipy fsolve (version 1.9.1) was used. In a second step, fsolve was used to invert this calculation and find values of an additional filtration ${t}_{\mathrm{ref}}$ to achieve a required half-value layer ${HVL}_{\mathrm{ref}}$ for the corresponding RQR standard radiation quality (e.g. 1.42 mm Al for RQR 2), solving the following equation:


(6) \begin{equation*} {\left({HVL}_{\mathrm{ref}}- HVL\Big({K}_{\mathrm{air},\kern0.1em\mathrm{att}}\left({t}_{\mathrm{ref}}\right)\right)}^2=0 \end{equation*}

For validation, Equation 6 was used to calculate the quantity ${K}_{\mathrm{air}}/{K}_{\mathrm{air},\mathrm{att}}\left({t}_{\mathrm{ref}}\right)$, which is required to lie within the interval $\left[0.485,0.515\right]$, this being the validation criterion specified in IEC 62167^(^^4^^)^. In addition, the homogeneity coefficient $h$ was calculated, which is the ratio of the first to second $HVL$ and is expected to lie within ±$0.03$ of the value given in IEC 61267^(^^4^^)^.

### Ranges of variation for influence quantities

For the establishment of the radiation qualities defined in IEC 61267^(^^4^^)^, several conditions regarding the anode angle of the X-ray tube, the practical peak voltage ($PPV$) and the achieved $HVL$ must be met within certain limits. These limits were chosen to define ranges of variation for each influence quantity (anode angle, $PPV$, ${HVL}_{\mathrm{ref}}$), and ${SNR}_{\mathrm{in}}^2$ was investigated within these ranges.

The anode angle must exceed ${9}^{{}^{\circ}}$ to allow the establishment of radiation qualities as defined within the standard. IEC 61267^(^^4^^)^ makes no recommendation concerning maximum values of the anode angle. For this work, the maximum value was set to ${30}^{{}^{\circ}}$ to cover a typical range.

$PPV$ must be set within an uncertainty ($k=2$) of $1.5\%$ or $1.5\ \mathrm{kV}$, whichever is larger. For the simulations, the X-ray tube voltage was altered between $-2.25$ and $2.25\ \mathrm{kV}$, representing a conservative range estimate.

For the establishment of RQR radiation qualities in accordance with IEC 61267^(^^4^^)^ the established radiation quality must be verified using a HVL test device. The quotient of the air kerma reading with the HVL test device and the air kerma reading without the HVL test device ($K/{K}_0$) must lie within the range of $0.485$ and $0.515$. This range was realised by varying ${HVL}_{\mathrm{ref}}$ between $-5$ and $5\%$ relative to the value given in IEC 61267^(^^4^^)^.

### Mean values, standard deviation and coverage intervals

To quantify the range of ${SNR}_{\mathrm{in}}^2$ for a specific radiation quality that allows a certain range for $PPV$, $HVL$ and anode angle, an estimate of ${SNR}_{\mathrm{in}}^2$ was calculated by Monte Carlo methods following the procedure described in the Guide to the Expression of Uncertainty in Measurement^(^^9^^)^. For this, the variations of $PPV$, ${HVL}_{\mathrm{ref}}$ and anode angle were drawn from rectangular distributions and limited by the previously described variation limits.

## Results

### Influence of anode angle

**Figure 1:**
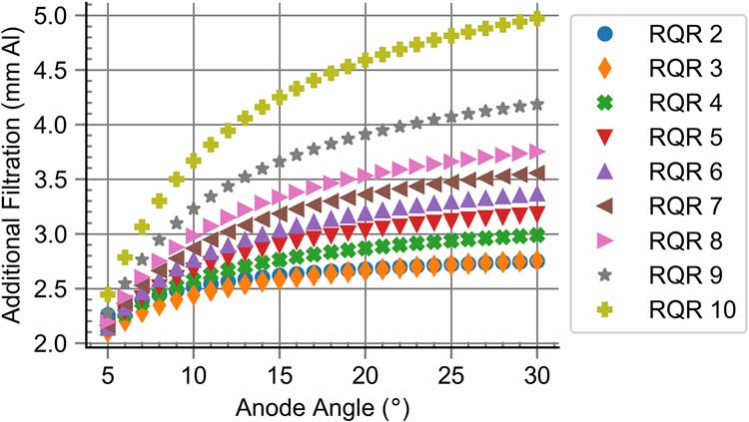
Additional filtration needed for the realisation of RQR standard radiation qualities for different anode angles.

Figure 1 shows the additional filtration needed for the realisation of RQR radiation qualities for different anode angles. It can be observed that the additional filtration needed increased along with the anode angle. Applying these additional filtrations, the difference between the target $HVL$ and the obtained $HVL$, $\Delta HVL$, was <$0.001\ \mathrm{mm}\ \mathrm{Al}$ for all standard radiation qualities and anode angles. The difference between the target homogeneity coefficient and the obtained homogeneity coefficient, $\Delta h$, was <$0.022$ for all standard radiation qualities and anode angles; deviations from $\Delta h=0$ increased for low anode angles. The simulation of the HVL test device resulted in the values $K/{K}_0$, which were within the interval $\left[0.485,0.515\right]$ for all anode angles.

**Figure 2:**
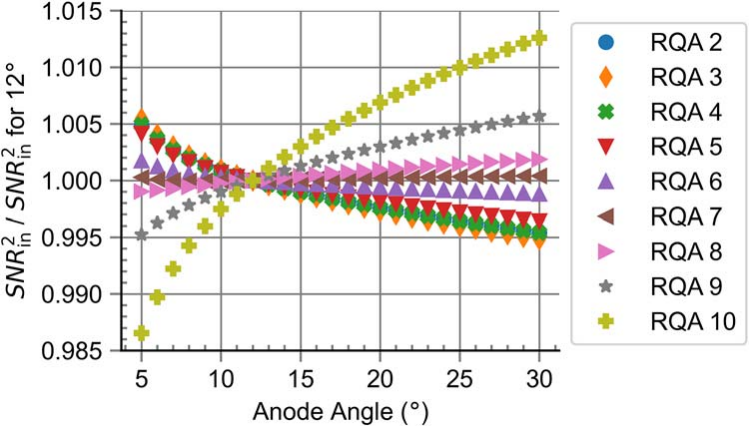
Influence of different anode angles on the ${SNR}_{\mathrm{in}}^2$ values for the computational realisation of RQA standard radiation qualities, referenced to an anode angle of 12°.

Figure 2 shows the influence of different anode angles on the ${SNR}_{\mathrm{in}}^2$ values for the computational realisation of RQA standard radiation qualities, referenced to an anode angle of ${12}^{{}^{\circ}}.$ For high and low energies, the influence of the anode angle was >$5\%$ for both low and high anode angles. Only the radiation qualities RQA 7 to RQA 9 were roughly stable (within $\pm 0.5\%$) over the range of anode angles. For radiation qualities with larger mean energies, ${SNR}_{\mathrm{in}}^2$ increased with anode angle. For RQA 6 and lower energies, ${SNR}_{\mathrm{in}}^2$ decreased with increasing anode angle.

### Influence of energy

**Figure 3:**
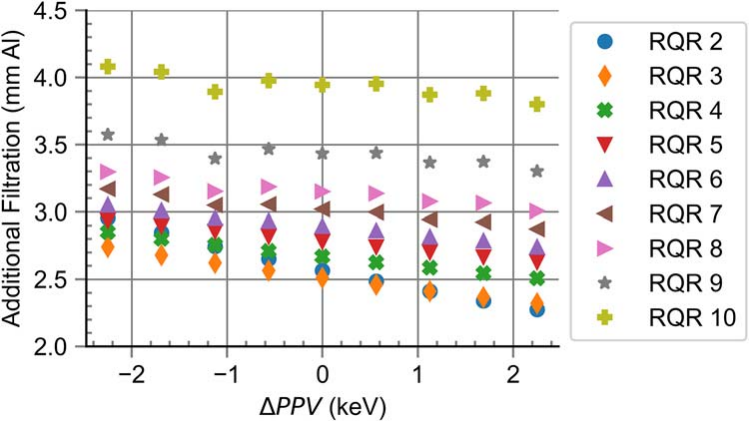
Additional filtration needed for the realisation of RQR standard radiation qualities at different X-ray tube voltage deviation levels.

Figure 3 shows the results of the computational realisation of RQR radiation qualities for different deviations in X-ray tube voltage relative to the optimal value, $\Delta PPV$. It was observed that the additional filtration needed decreased linearly with $\Delta PPV$. Applying these additional filtrations, the difference between the target $HVL$ and the obtained $HVL$, $\Delta HVL$, was <$0.001\ \mathrm{mm}\ \mathrm{Al}$. The difference between the target homogeneity coefficient and the obtained homogeneity coefficient, $\Delta h$, was <$0.022$ for all X-ray tube voltages under investigation. The simulation of the HVL test device showed the values $K/{K}_0$ lying within the interval $\left[0.485,0.515\right]$ for all anode angles.

**Figure 4:**
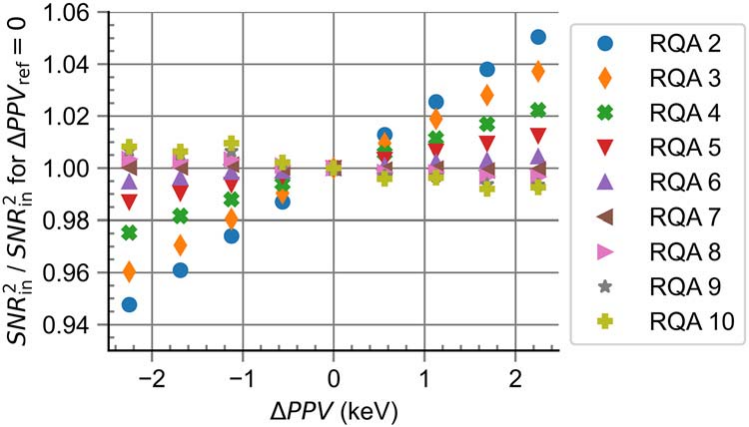
Influence of different values of $\Delta PPV$ on the ${SNR}_{\mathrm{in}}^2$ values for the computational realisation of RQA standard radiation qualities.

Figure 4 shows the influence of $\Delta PPV$ on the ${SNR}_{\mathrm{in}}^2$ values for the computational realisation of RQA standard radiation qualities. The influence of $\Delta PPV$ was larger for radiation qualities with low mean energies, with a maximum deviation of $>4\%$ for RQA 2 for low and high values of $\Delta PPV$. This effect decreased for standard radiation qualities with larger mean energies up to RQA 7, where the influence of a change in the tube voltage was < $0.1\%$. For RQA 8 and higher, the influence of $\Delta PPV$ again increased, with a maximum deviation of $1\%$ seen for RQA 10 at the largest and smallest $\Delta PPV$ values.

### Influence of HVL

**Figure 5:**
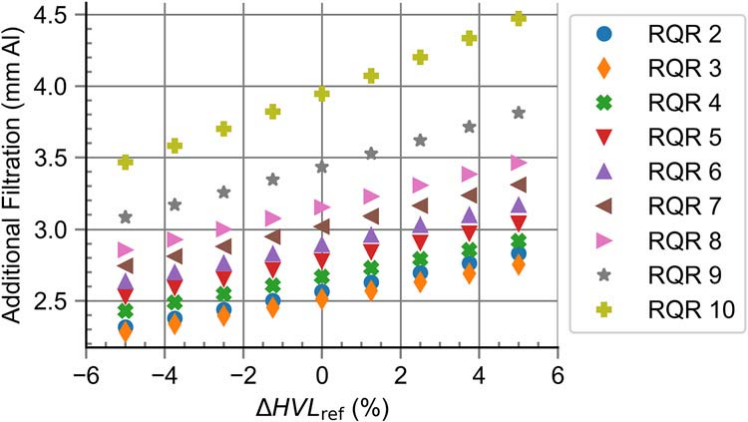
Additional filtration needed for the realisation of RQR standard radiation qualities for different values of HVL_ref_ relative to the optimal value.

Figure 5 shows the results of the computational realisation of RQR radiation qualities for different deviations in ${HVL}_{\mathrm{ref}}$ relative to the optimal value, $\Delta{HVL}_{\mathrm{ref}}$. It can be observed that the additional filtration needed increased linearly with $\Delta{HVL}_{\mathrm{ref}}$. The difference between the target homogeneity coefficient and obtained homogeneity coefficient, $\Delta h$, was <$0.03$ for all $\Delta{HVL}_{\mathrm{ref}}$ under investigation. The simulation of the HVL test device showed the values $K/{K}_0$ lying within the interval $\left[0.485,0.515\right]$ for all anode angles.

**Figure 6:**
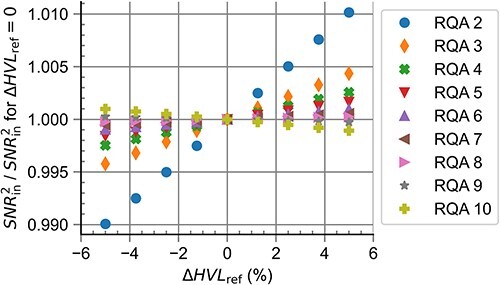
Influence of different values of ΔHVL_ref_ on the ${SNR}_{in}^2$ values for the computational realisation of RQA standard radiation qualities.

Figure 6 shows the influence of $\Delta{HVL}_{\mathrm{ref}}$ on the ${SNR}_{\mathrm{in}}^2$ values for the computational realisation of RQA standard radiation qualities. The influence of $\Delta{HVL}_{\mathrm{ref}}$ was larger for radiation qualities with lower mean energies, with a maximum deviation of > $1\%$ for RQA 2, but decreased drastically radiation qualities with larger mean energies and was below $0.1\%$ for RQA 6 and above.

### Maximum and minimum values for ${\boldsymbol{SNR}}_{\mathbf{in}}^{\mathbf{2}}$ within a radiation quality

Tables 2 and 3 show the maximum and minimum values for ${SNR}_{\mathrm{in}}^2$ within RQA standard radiation qualities. Maximum values for ${SNR}_{\mathrm{in}}^2$ were achieved by choosing the smallest anode angle possible and a maximum increase in $PPV$ and by optimising for a larger value for ${HVL}_{\mathrm{ref}}$ for radiation qualities up to RQA 6. The opposite was observed for minimum values. Deviations between the minimum and maximum values decreased up to RQA 7, where the parameters under investigation had almost no influence on ${SNR}_{\mathrm{in}}^2$. For radiation qualities with larger mean energies, the influence of the parameters under investigation changed. Larger anode angle paired with smaller $PPV$ and ${HVL}_{\mathrm{ref}}$ values led to maximum values for ${SNR}_{\mathrm{in}}^2$. By contrast, lower anode angle paired with larger $PPV$ and ${HVL}_{\mathrm{ref}}$ values resulted in minimum values for ${SNR}_{\mathrm{in}}^2$. Generally speaking, the spread of ${SNR}_{\mathrm{in}}^2$ was large for radiation qualities with small mean energies and became less for radiation qualities with larger mean energies.

**Table 2:** Maximum values for ${SNR}_{\mathrm{in}}^2$ for each RQA standard radiation quality, including the corresponding parameters for the anode angle, $\Delta PPV$ and $\Delta{HVL}_{\mathrm{ref}}$. $\Delta$min describes the percentual deviation to the minimum value of ${SNR}_{\mathrm{in}}^2$ within the same standard radiation quality (cf. Table 3).

Radiation quality	$\mathrm{Anode}\ \mathrm{angle}$ [[ineq163]][°]	$\Delta PPV\ \left[\mathrm{kV}\right]$	$\Delta{HVL}_{\mathrm{ref}}$ [%]	Max. ${SNR}_{\mathrm{in}}^2\ \left[{\mathrm{mm}}^{-2}\ \mathrm{\mu} \mathrm{Gy}\right]$	$\Delta$ min [%]
RQA 2	9	2.25	5	15 026	13.8
RQA 3	9	2.25	5	21 862	9.7
RQA 4	9	2.25	5	27 156	6.0
RQA 5	9	2.25	5	30 452	3.4
RQA 6	9	2.25	5	32 264	1.3
RQA 7	30	0	5	32 830	0.24
RQA 8	30	−2.25	5	32 872	0.96
RQA 9	30	−2.25	−5	31 586	2.1
RQA 10	30	−2.25	−5	28 565	3.5

**Table 3:** Minimum values for ${SNR}_{in}^2$ for each RQA standard radiation quality, including the corresponding parameters for the anode angle, $\Delta PPV$ and $\Delta{HVL}_{ref}$. $\Delta$max describes the percentual deviation to the maximum value of ${SNR}_{in}^2$ within the same standard radiation quality (cf. Table 2).

Radiation quality	$\mathrm{Anode}\ \mathrm{angle}$ [°]	$\Delta PPV\ \left[\mathrm{kV}\right]$	$\Delta{HVL}_{\mathrm{ref}}$ [%]	Min. ${SNR}_{\mathrm{in}}^2\ \left[{\mathrm{mm}}^{-2}\ \mathrm{\mu} \mathrm{Gy}\right]$	$\Delta$ max [%]
RQA 2	30	−2.25	−5	13 206	−13.8
RQA 3	30	−2.25	−5	19 933	−9.7
RQA 4	30	−2.25	−5	25 621	−6.0
RQA 5	30	−2.25	−5	29 447	−3.4
RQA 6	30	−2.25	−5	31 835	−1.3
RQA 7	9	2.25	−5	32 751	−0.24
RQA 8	9	2.25	−5	31 586	−0.96
RQA 9	9	2.25	5	30 930	−2.1
RQA 10	9	2.25	5	27 602	−3.5

### Standard deviation and coverage intervals

**Figure 7:**
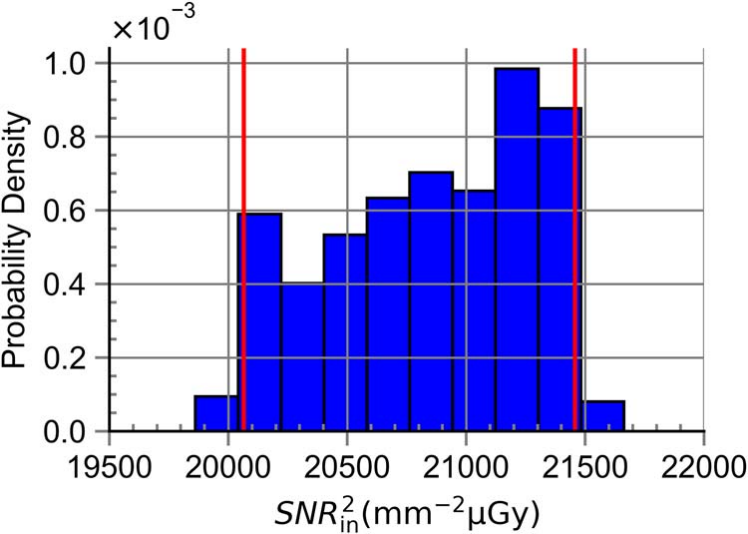
Probabilistic distribution of ${SNR}_{\mathrm{in}}^2$ for the full range of anode angle, ΔPPV and ΔHVL_ref_ for the RQA 3 standard radiation quality. This distribution resulted in the largest value for u(y) within this study.

**Figure 8:**
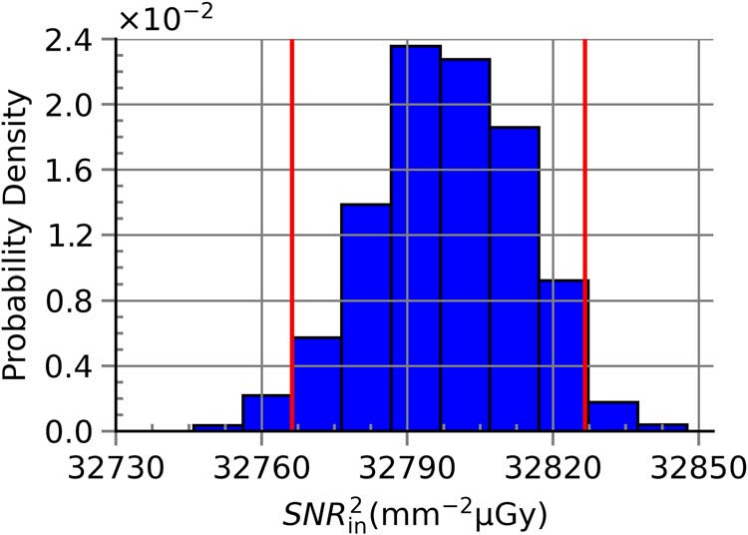
Probabilistic distribution of ${SNR}_{\mathrm{in}}^2$ for the full range of anode angle, ΔPPV and ΔHVL_ref_ for the RQA 7 standard radiation quality. This distribution resulted in the largest value for u(y) within this study.

**Figure 9:**
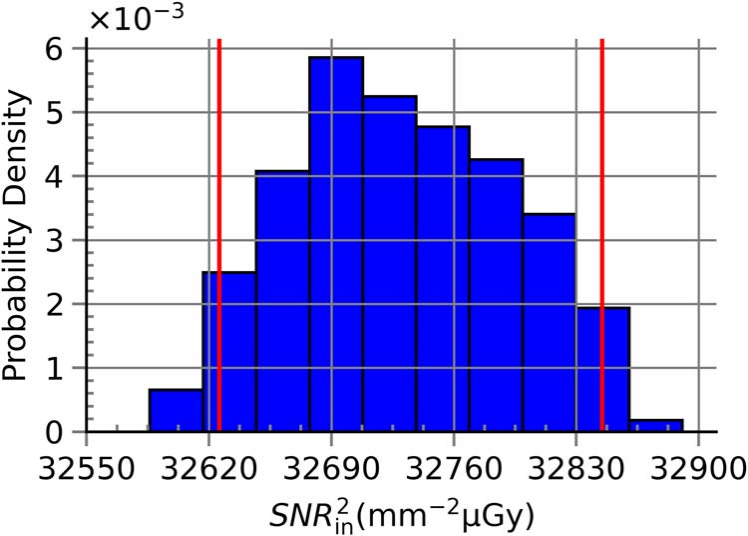
Probabilistic distribution of ${SNR}_{\mathrm{in}}^2$ for the full range of anode angle, ΔPPV and ΔHVL_ref_ for the RQA 8 standard radiation quality. This distribution resulted in the largest value for u(y) within this study.

**Figure 10:**
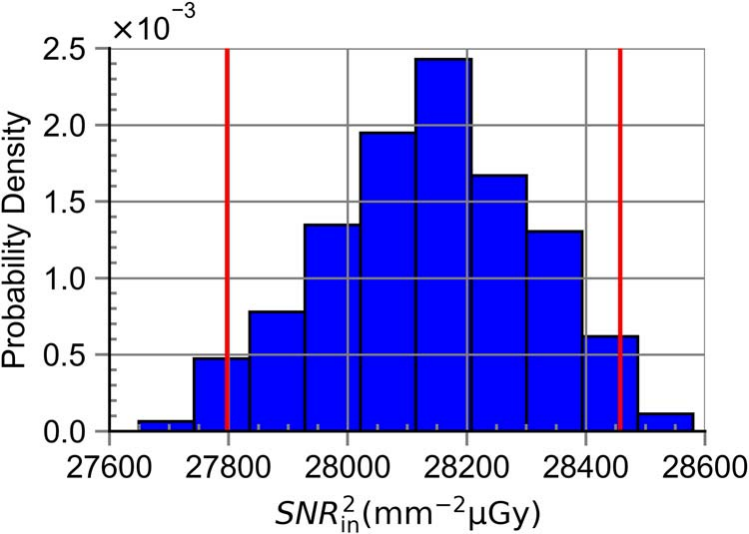
Probabilistic distribution of ${SNR}_{\mathrm{in}}^2$ for the full range of anode angle, ΔPPV and ΔHVL_ref_ for the RQA 10 standard radiation quality. This distribution resulted in the largest value for u(y) within this study.

Table 4 shows simulated ${SNR}_{\mathrm{in}}^2$ values, $u(y)$ and the probabilistically symmetric 95% coverage interval for all RQA standard radiation qualities. The results here confirmed the results presented in Tables 2 and 3. The largest values for $u(y)$ and the coverage intervals were seen for RQA 3 ($u(y)=432$), after which $u(y)$ decreased with energy up to RQA 7 ($u(y)=16$) before exhibiting a slight increase that did not exceed $u(y)=168$. Figures 7–10 show the obtained probabilistic distributions of ${SNR}_{\mathrm{in}}^2$ over the parameters of interest for selected RQA standard radiation qualities. The distributions were shifted to larger values (compared with a normal distribution) for photon fluences with lower mean energies and became more and more shifted to lower values for radiation qualities with larger mean energies (RQA 8). For the high energies, the distribution became more symmetric (RQA 10).

**Table 4:** ${SNR}_{\mathrm{in}}^2$ values, u(y) and the probabilistically symmetric 95% coverage interval for all RQA standard radiation qualities.

Radiation quality	${SNR}_{\mathrm{in}}^2\ \left[{\mathrm{mm}}^{-2}\ \mathrm{\mu} \mathrm{Gy}\right]$	$u(y)$	Probabilistically symmetric 95% coverage interval	Percentage change to IEC 62220-1(1–3)
RQA 2	14 050	390	[13 313, 14 643]	-
RQA 3	20 829	432	[20 015, 21 460]	−4.27%
RQA 4	26 340	337	[25 700, 26 834]	-
RQA 5	29 921	214	[29 516, 30 243]	−0.84%
RQA 6	32 056	89	[31 882, 32 188]	-
RQA 7	32 798	16	[32 766, 32 831]	1.35%
RQA 8	32 735	63	[32 628, 32 853]	-
RQA 9	31 302	125	[31 071, 31 542]	0.72%
RQA 10	28 153	168	[27 800, 28 462]	-

## Discussion

The results have shown that the computational optimizer succeeded in finding a value for the additional filtration that, in turn, led to the targeted value for the quotient of the air kerma reading without and the air kerma reading with the HVL test device (0.485–0.515). It can therefore be concluded that the RQA qualities that were simulated based on this additional filtration were well suited to investigating the robustness of ${SNR}_{\mathrm{in}}^2$. This conclusion is also justified by the observation that $\Delta h$ resulted in values <0.03 for all investigations, which is a requirement specified by TRS-457^(^^10^^)^.

In establishing RQR standard radiation qualities, the purpose of the additional filtration is to introduce beam hardening in order to achieve a specific value for ${HVL}_{\mathrm{ref}}$. As such, an increase in the needed additional filtration points to a decrease in the energy of the photons. This can be directly observed in Figure 3, which reveals that a lower value for $PPV$ was linked to an increase in the amount of additional filtration needed.

Changing the anode angle also has an influence on the distribution of the photon fluence with respect to the energy. A small anode angle leads to an anode that is more perpendicular to the electron beam, meaning that the photons produced in the beam direction will be affected by a beam hardening effect of the anode itself and therefore have a larger energy. In contrast, high anode angles lead to smaller energies and, as described above, to an increase in needed additional filtration, which explains the effect seen in Figure 1.

As a beam specifier, $HVL$ increases with the mean energy for a given photon fluence. Therefore, lowering the target $HVL$ (${HVL}_{\mathrm{ref}}$) leads to a target fluence with a lower mean energy, so less additional filtration is needed for a lower value of ${HVL}_{\mathrm{ref}}$, cf. Figure 5.

Comparing the results obtained for the expected value of ${SNR}_{\mathrm{in}}^2$ to the values given in IEC 62220-1 ^(^^1–3^^)^ (Table 4), the deviations exceed the probabilistically symmetric 95% coverage interval for RQA 3 and RQA 7 but not the limits of the distribution shown in Tables 2 and 3. Further investigations on this difference were not possible because the methods for calculating the current values are not described in the literature.

Figures 2, 4 and 6 show that the high values for $u(y)$ observed for radiation qualities with low mean energies are not so much influenced by the anode angle but more by the limitation linked to the uncertainty of $PPV$ measurements and the allowed range for deviations from ${HVL}_{\mathrm{ref}}$. This demonstrates the necessity for strict requirements regarding the uncertainty of $PPV$ measurements in the context of establishing standard radiation qualities. Such requirements can be met through the use of invasive X-ray tube voltage measurements.

The energy dependence of $u(y)$ is caused by $\frac{\mu_{\mathrm{tr}}}{\rho }(E)$, which increases greatly when moving from larger to lower photon energies, thus directly influencing the related value for air kerma, ${K}_{\mathrm{air}}$. Therefore, a small variation of the mean energy for a photon fluence with a lower mean energy leads to a larger change of ${K}_{\mathrm{air}}$ and therefore to a broader histogram. For photon fluences with larger mean energies, this effect diminishes due to the lessening of the overall contribution to ${K}_{\mathrm{air}}$ of the low energy part of the photon fluence.

To derive an uncertainty budget for $DQE$, additional uncertainties must be considered. The uncertainty of a typical dose measurement in a secondary standard laboratory is given in TRS-457^(^^10^^)^. The scenario described in TRS-457^(^^10^^)^ should be a reasonable approximation to the situation at a testing laboratory. For a typical calibration setup and a reference class ionisation chamber, TRS-457 estimates a relative combined standard uncertainty of the reference value of the air kerma of $1.57\%$  $\left(k=1\right)$^(^^10^^)^. For the measurement of the modulation transfer function, IEC 62220-1-3^(^^3^^)^ allows a maximum variation of 5%. The uncertainty linked to the determination of the modulation transfer was discussed in detail in literature^(^^11^^)^. For the determination of the noise power spectrum and lag effects, IEC 62220-1-3 gives methods with an accuracy of better than 5%^(^^3^^)^.

## Conclusions

Even when established in accordance with the requirements described in IEC 61267^(^^4^^)^, a single standard radiation quality established in different laboratories can still reveal drastic differences in ${SNR}_{\mathrm{in}}^2$, especially for low energies. So for one and the same radiation quality, this variance can translate into a significant discrepancy in the number of photons per air kerma arriving at an imaging detector under investigation.

This complicates the comparison of different imaging detectors based on $DQE$ and must be considered when calculating uncertainties related to $DQE$ and when comparing different imaging detectors based on this quantity. The most important parameter for improving this situation is the uncertainty associated with $PPV$ measurements.
